# EOS^®^ biplanar X-ray imaging: concept, developments, benefits, and limitations

**DOI:** 10.1007/s11832-016-0713-0

**Published:** 2016-02-16

**Authors:** Elias Melhem, Ayman Assi, Rami El Rachkidi, Ismat Ghanem

**Affiliations:** Department of Orthopaedic Surgery, Hôtel-Dieu de France Hospital, University of Saint Joseph, Boulevard Alfred Naccache, Achrafieh, P.O. Box 166830, Beirut, Lebanon; Laboratory of Biomechanics and Medical Imaging, Faculty of Medicine, University of Saint Joseph, Beirut, Lebanon

**Keywords:** EOS radiography, Biplanar X-rays, Sagittal balance, Lower limbs, Scoliosis, Biomechanics

## Abstract

**Purpose:**

In 1992, Georges Charpak invented a new type of X-ray detector, which in turn led to the development of the EOS^®^ 2D/3D imaging system. This system takes simultaneous anteroposterior and lateral 2D images of the whole body and can be utilized to perform 3D reconstruction based on statistical models. The purpose of this review is to present the state of the art for this EOS^®^ imaging technique, to report recent developments and advances in the technique, and to stress its benefits while also noting its limitations.

**Methods:**

The review was based on a thorough literature search on the subject as well as personal experience gained from many years of using the EOS^®^ system.

**Results:**

While EOS^®^ imaging could be proposed for many applications, it is most useful in relation to scoliosis and sagittal balance, due to its ability to take simultaneous orthogonal images while the patient is standing, to perform 3D reconstruction, and to determine various relationships among adjacent segments (cervical spine, pelvis, and lower limbs). The technique has also been validated for the study of pelvic and lower-limb deformity and pathology in adult and pediatric populations; in such a study it has the advantage of allowing the measurement of torsional deformity, which classically requires a CT scan.

**Conclusions:**

The major advantages of EOS^®^ are the relatively low dose of radiation (50–80 % less than conventional X-rays) that the patient receives and the possibility of obtaining a 3D reconstruction of the bones. However, this 3D reconstruction is not created automatically; a well-trained operator is required to generate it. The EOS^®^ imaging technique has proven itself to be a very useful research and diagnostic tool.

## Introduction

**Fig. 1:**
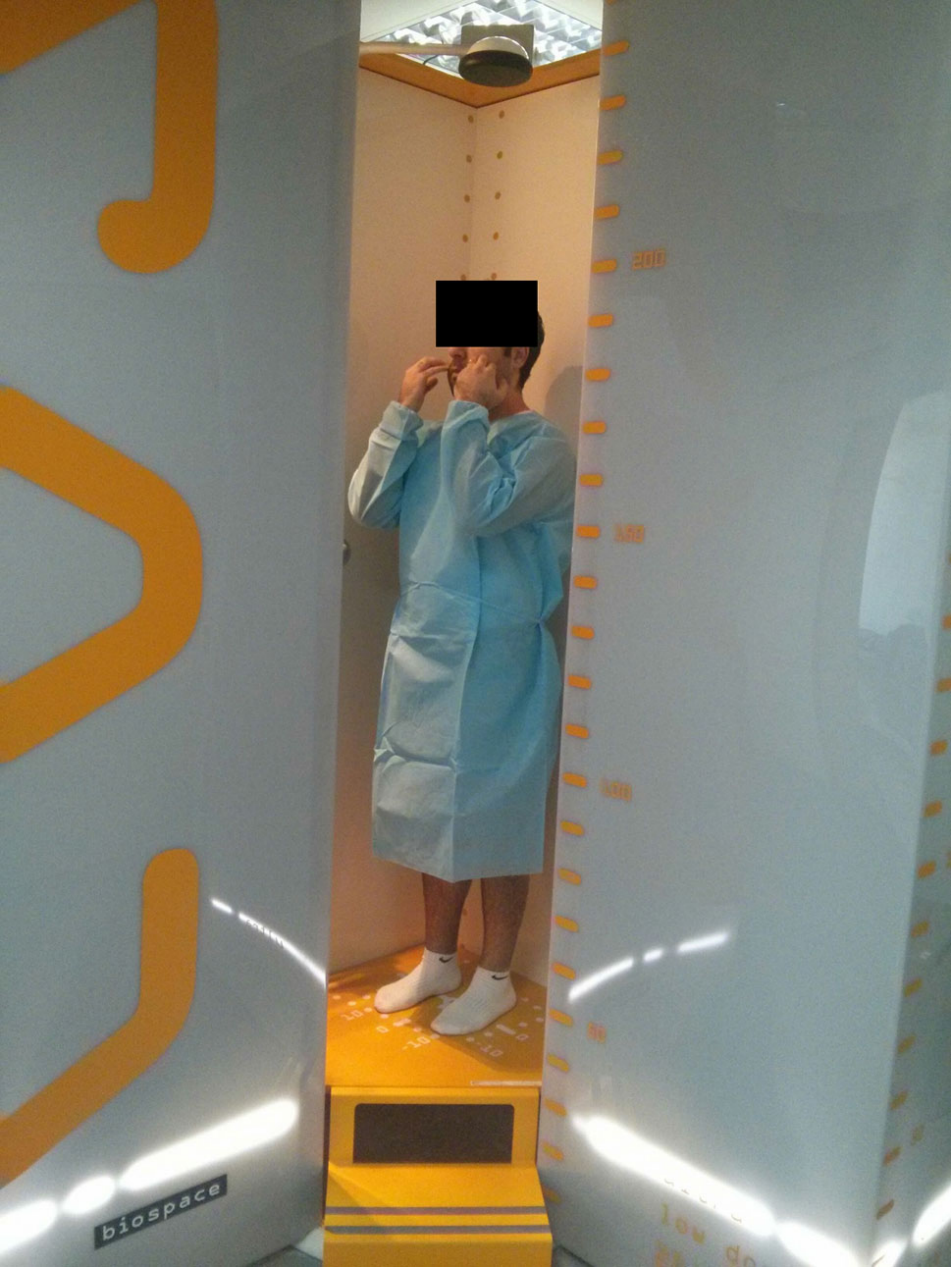
A patient standing in the EOS^®^ cabin in the required position for acquisition of orthogonal radiographs

In 1992, Professor Georges Charpak received the Nobel Prize in Physics for his invention of a gaseous particle detector with a multiwire proportional chamber [1]. This invention led to the development of the EOS^®^ 2D/3D imaging system, which uses this ultrasensitive multiwire proportional chamber detector to detect X-rays, thus limiting the dose of X-rays that must be absorbed by the patient. The EOS^®^ system also allows simultaneous anteroposterior (AP) and lateral 2D images of the whole body to be taken in a calibrated environment, permitting the 3D reconstruction of spine and lower limb bony structures by stereoradiography [2–4]. The images are taken in the standing position, allowing the spine and lower limbs to be examined under normal weight-bearing conditions. Other publicized advantages of EOS^®^ imaging include true-to-size images, since the machine scans the body with two 45-cm-wide X-ray beams, unlike the single-source divergent X-ray beam in conventional radiology, which induces magnification of the image (Fig. 1). The EOS^®^ system scans the body in 10–25 s [2]. Image quality was found to be comparable to X-ray imaging, and the radiation dose was significantly lower (0.07 mGy for the PA spine, as compared to 0.92 mGy) [5, 6]. More recently, EOS Imaging^®^ has launched a new feature on its machine: a microdose protocol that reduces the dose to the patient 5.5-fold [7].

The sterEOS^®^ software bundled with the EOS^®^ imaging system makes it possible to perform 3D reconstruction of bone structures. This software uses algorithms based on statistical modeling and bone shape recognition [3, 4]. A well-trained operator can perform 3D reconstruction of the skeletal envelope, and about 100 clinical parameters for the spine and lower limbs are automatically calculated. Advantages include the ability to measure 3D angles and dimensions, unaffected by differences in projection and image acquisition, as well as the capacity to determine torsion angles which usually require CT scan images and cannot be adequately calculated with conventional 2D radiology. The EOS^®^ system is mainly an imaging tool for the skeleton, as it is based on X-rays. However, although it is usually compared to CT-scan images due to its ability to provide 3D reconstruction, it does not provide information on soft tissues such as muscles, spinal cord, nerves, and viscera.

Its range of applications in daily practice is growing exponentially, although they mainly focus on spine, pelvis, and lower-limb imaging. The purpose of this review is to present the state of the art for EOS^®^ imaging, to report on recent developments and advances in this technique, and to stress its benefits while also noting its limitations, based on a thorough literature search on the subject as well as personal experience supported by illustrative case reports.

## Image quality and radiation dose

### Image quality

In order to study the quality of the images obtained by the EOS^®^ technique, Deschênes et al. compared EOS^®^ images to computed radiography (CR) in 50 patients requiring spine radiographs [8]. The quality comparison was done using a quantitative assessment questionnaire that was completed by two radiologists and two blinded orthopedic surgeons after they had analyzed the radiographs. The EOS^®^ system was found to be superior or equivalent to CR in terms of global image quality and structure visibility in 97.2 and 94.3 % of images, respectively. Visibility in EOS^®^ images was significantly better for all structures in the PA view (*p* = 0.006) and sagittal view (*p* = 0.037) whereas the lumbar spinous processes were more visible on CR (*p* = 0.003). The authors attributed this to technical limitations imposed by the study design, where EOS^®^ images were adjusted to be the same quality as CR in the thoracolumbar junction, leading to poorer results in the lumbar region. On the other hand, in a comparison of quality between EOS^®^ 2D and conventional X-ray images in pelvis and knee examinations of 114 patients [9], as determined by four independent radiologists, standard X-ray images were deemed superior in 83 % of comparisons and EOS^®^ 2D images in only 2 % of cases; 30 % of the EOS^®^ 2D images were considered diagnostically inaccurate compared with 0.8 % of the conventional X-ray images. The authors concluded that EOS^®^ biplanar lower limb X-ray is not suitable for the diagnostic assessment of bone morphology of the lower limb. Although we fully agree that printed images given by the EOS^®^ system are less bright than conventional 2D radiographs, we have been using EOS^®^ imaging for over 4 years now without any specific diagnostic difficulties compared to conventional radiographs. Image quality was also assessed on the cervical spine. EOS^®^ radiographs were found to have good intraobserver repeatability and interobserver reproducibility (difference ≤0.54 mm for length measurements and ≤0.33° for angle measurements) in determining the shapes and positions of lower cervical spine vertebrae [10].

### Radiation dose

There are two acquisition protocols for the EOS^®^ imaging technique: the standard low-dose protocol and the microdose protocol.

In Deschênes’ previously mentioned study [8], the entrance skin dose was measured using skin dosimeters and found to be three times lower for the nape of the neck and six to nine times lower for the thoracolumbar region when the EOS^®^ system was used rather than conventional X-rays. For femoral and tibial torsion measurements, the radiation dose for EOS^®^ 3D reconstruction was 4.1 times lower to the ovaries, 24 times lower to the testicles, and 13–30 times lower to the knees and ankles as compared to a CT scan [11].

In an attempt to further decrease the radiation dose, especially in children and cases requiring repeated radiographs for clinical follow-up, EOS Imaging^®^ developed a new microdose protocol that can now be added to any existing machine. However, there are currently no clinical studies that evaluate the radiation dose provided with this microdose technique, given that it is still a new development. Nonetheless, EOS Imaging^®^ specifies that the microdose delivers 5.5 times less radiation than the usual low-dose protocol, and 45 times less radiation than conventional radiography.

## Spine

Multiple studies have proposed different manual and semi-automated methods for reconstructing the spine from two 2D orthogonal images [3, 12–14]. These have largely formed the basis of the 3D reconstruction of the spine using the sterEOS^®^ software.

### Scoliosis and sagittal balance

**Fig. 2:**
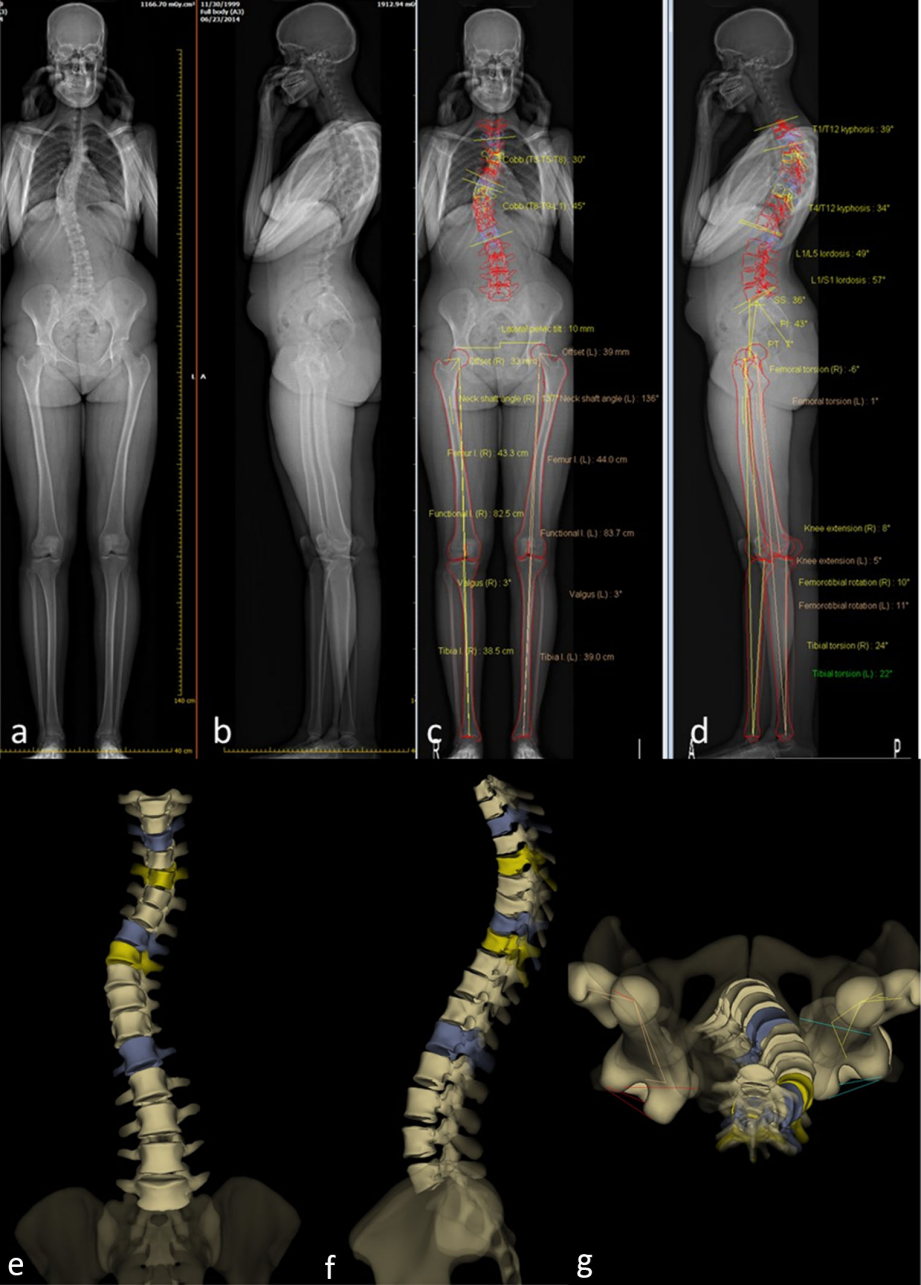
Example of 2D orthogonal X-ray images for a 15-year-old patient obtained using the EOS^®^ system, along with a 3D reconstruction

While EOS^®^ imaging could be proposed for many applications, the two domains for which it is the most useful are scoliosis and sagittal balance [15], given that it allows simultaneous orthogonal images to be taken while standing, as well as 3D reconstruction (Fig. 2). Different authors have evaluated the accuracy and precision of radiological parameters measured from 2D radiographs and 3D reconstructions obtained by the EOS^®^ imaging technique.

#### Accuracy, reproducibility, and reliability of EOS measurements

*Precision of 2D measurements* In a study that included the cervical spine, Vidal et al. [16] evaluated the interobserver reproducibility and intraobserver repeatability for 11 spinal measurements on 50 AIS patients and 25 normal controls using EOS^®^ 2D X-ray images. Those included the angles C1–C3, C2–C6, C3–C7, T1–T12, and L1–L5, the C7 plumb line (C7PL), the external auditory canal plumb line (CAEPL), external auditory canal–hips (CAEH), and the external auditory canal plumb line to the C7 plumb line (CAEC7). All of these parameters showed excellent interobserver reproducibility and intraobserver repeatability (ICC > 0.8).

*2D vs 3D EOS*^*®*^ Somoskeoy et al. evaluated the accuracy and reliability of spinal measurements made using EOS^®^ 3D reconstruction in 201 individuals (10 healthy controls and 191 patients with spinal deformities, the majority of whom had adolescent idiopathic scoliosis), as compared to 2D measurements [17]. EOS^®^ 3D measurements had very high intraobserver repeatability for Cobb angle, thoracic kyphosis, and lumbar lordosis, and better interobserver reproducibility than 2D methods. There were no statistically significant differences between EOS^®^ 3D and 2D manual measurements except for lumbar lordosis, where a 2° difference was found.

*EOS*^*®*^*3D reconstruction vs CT scan* Al-Aubaidi et al. compared measurements of apical vertebral orientation (AVO) between EOS^®^ 3D reconstructions and CT-scan 3D reconstructions in seven patients with scoliosis [18]. There was no statistically significant difference in intra- or interobserver reliability for the measurement of AVO between EOS^®^ and 3D CT-scan, and both yielded similar measurements. However, unlike CT-scan 3D reconstruction, variations in patient positioning (i.e., not strictly facing the source, with a slight rotation) may possibly impact the accuracy and precision of EOS^®^ 3D reconstruction. To account for this, three scoliotic spine phantoms were scanned at 0°, ±5°, and ±10° in the upright position using the EOS^®^ system and in the horizontal position using CT [19]. The differences between the EOS^®^ system and CT in the positions, orientations, and shapes of vertebrae were small; the root mean square (RMS) of the offset (maximal vertebral translation) was 1.2 mm and the RMS of axial rotation was 1.94°. There was also a very small difference in shape, with a RMS error of 1.32 mm. The pedicles were the most poorly modeled anatomical structure in the EOS^®^ reconstruction as compared with CT scan (*p* < 0.05), and the thoracic spine had better shape accuracy than the lumbar spine. The mean differences for radiological parameters were 1° for pelvic incidence, <1° for kyphosis and lordosis, and 1.6° for Cobb angle. Axial rotation of the phantom between −10° and +10° did not significantly affect the results. Patient malpositioning in the ±10° range was therefore shown to be acceptable. Furthermore, Ilharreborde et al. [7] evaluated the precision of the 3D reconstruction of radiographs using the EOS^®^ microdose protocol in 32 adolescent idiopathic scoliosis (AIS) patients. They found it to have excellent repeatability and reproducibility (ICC > 0.92) for all relevant spinal and pelvic parameters.

*Validation in the presence of implants* Since orthopedic implants might affect the 3D reconstruction of the spine, Ilharreborde et al. [20] measured the repeatability and reproducibility of radiological parameters obtained from EOS^®^ 3D reconstruction for preoperative and postoperative evaluation of AIS patients. For preoperative measurements, the interobserver and intraobserver reproducibility were excellent for spinal parameters and pelvic parameters. For postoperative measurements, interobserver reproducibility was good for spinal parameters and excellent for pelvic parameters, while the intraobserver repeatability was excellent. The difference between pre- and postoperative interobserver reproducibility was less than 1° for all parameters except for the apical vertebral rotation (AVR) (4.3°). The authors attributed this to the presence of implants on the apex of scoliotic curves that interfere with adequate determination of anatomical landmarks in the postoperative images.

#### Outcomes from 3D reconstruction

**Fig. 3:**
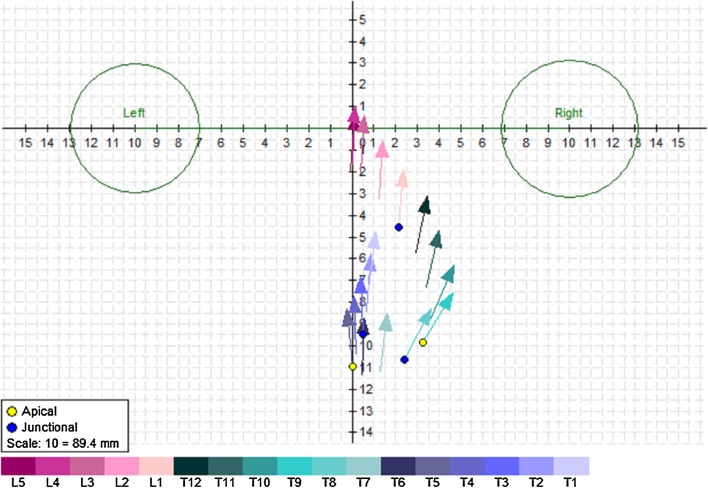
Example of the representation of spinal deformity using vertebral vectors

*Vertebra vectors* Taking advantage of the ability of the EOS^®^ system to visualize the spine in 3D, Illes et al. introduced a new concept: vertebra vectors [21]. Each vertebra vector starts from the midpoint of the interpedicular line, runs parallel to the upper endplate of the vertebra on the sagittal view and in the middle of the vertebral body on the axial view, and ends at the intersection of this line with the anterior surface of the vertebral body (Fig. 3). This allows any spinal deformity to be fully described using an understandable representation of the position, size, and rotation of each vertebra. By projecting the vertebra vectors, it is possible to calculate all of the parameters that define scoliosis which can be obtained from the 2D orthogonal images, and to determine the axial rotation. The concept of vertebra vectors was validated by comparing the calculated measurements to those obtained from 2D radiographs for the same set of patients [22]. It was also shown that the vertebra vector measurements were more reliable than 2D measurements when the scoliosis is severe [23].

*Scoliosis and thoracic cage geometry* The major life-threatening complication of scoliosis is its impact on the thoracic cage. The spinal penetration index (SPI) is a measure that quantifies the portion of the rib cage occupied by the vertebrae. It is usually determined using a CT scan in a supine position, which does not correspond to the standing SPI. Ilharreborde et al. obtained biplanar EOS^®^ radiographs in 80 AIS patients and performed a reconstruction of the spine and the rib cage [24]. This allowed the thoracic volume, the mean SPI (SPIm), which is the percentage of the thoracic cage volume occupied by vertebrae, and the apical SPI (SPIa), which is the percentage of the thoracic cage surface occupied by the apical vertebra, to be measured in an axial plane. However, this was not compared to the previously established gold standard (CT scan), and there is currently no direct practical application of the EOS^®^ system for pulmonary evaluation of spinal deformity patients. Moreover, 3D rib cage reconstruction using the EOS^®^ system is still not available for regular EOS^®^ users, as it is still under evaluation and therefore not commercialized.

In a later study, the same authors performed a 3D evaluation of the trunk following posterior instrumentation and fusion [25]. They performed EOS^®^ biplanar radiographs on 49 AIS patients preoperatively, in the early postoperative period, and at last follow-up. They reconstructed the spine and rib cage in 3D, and measured the thoracic volume, SPIm, SPIa, and the classic spinal and pelvic parameters. The authors showed that the thoracic volume increased the most with the correction of the apical vertebral rotation (AVR). This information would not have been available without EOS^®^ 3D reconstruction, as the excessive irradiation associated with CT scans limits the possibility of performing such studies due to ethical considerations.

Stereoradiographic modeling using the EOS^®^ system was evaluated in a study that aimed to determine the effect of the growing rod technique on rib cage shape [26]. The authors calculated its accuracy compared to CT scan in two of the patients, and found it to have good accuracy and excellent interobserver reproducibility and intraobserver repeatability for thoracic cage geometrical parameters.

*Assessment of vertebral wedging* Vertebral wedging has been theorized to be the major initiating deformity in scoliosis. Studies of its role and magnitude were previously limited by the high levels of radiation required to perform 3D reconstruction via CT scan, but they have recently been made possible by the EOS^®^ system. In a sample of 27 girls with AIS [27], wedging was calculated in three different planes for each vertebra. The authors found that wedging is present even in mild AIS, that it increases with the severity of the curve, and that it was most important in the three vertebrae immediately below the apex. The importance of studying vertebral wedging is still unknown, but it could impact decision-making in the future as our understanding of scoliosis improves.

*3D assessment of spine flexibility* One of the most important steps in classifying scoliosis in the preoperative setting is the determination of spinal flexibility. The most widely accepted method is lateral bending radiographs, but this is limited by patient cooperation and intertechnician variability. Therefore, Hirsch et al. [28] investigated a new standardized technique, the EOS^®^ suspension test, for evaluating spinal flexibility in 50 AIS patients scheduled for operation. In it, the patient is subjected to progressive traction forces through a rigid collar attached to cables until they are on tiptoes, at which level the EOS^®^ radiographs are taken. They compared the reduction achieved by this technique to that of the supine traction test on a Cotrel frame. Compared to the traction test, higher forces were applied during the EOS^®^ suspension test, and these forces were more standardized. However, the tolerance of the patient was lower in the EOS^®^ suspension test. The major advantage of this technique is the ability to analyze coronal, sagittal, and axial reduction in a single standardized setting.

*3D evaluation of brace treatment* In its early stages, scoliosis is usually treated by bracing. The correction of scoliotic deformity is usually studied by conventional 2D radiography, which only allows the evaluation of sagittal and coronal corrections. EOS^®^ 3D reconstruction allows axial deformity correction to be evaluated as well. Lebel et al. compared two types of braces used in 28 AIS patients: the thoracolumbosacral orthosis (TLSO) and the Chêneau-type brace [29]. Both braces offered similar sagittal and coronal corrections, but the Chêneau brace permitted greater correction of the AVR (8.2 vs 4.9°, *p* = 0.02), a finding that 2D radiography would not have been able to easily demonstrate.

*Scoliosis Research Society (SRS) 3D Committee* Given the importance of viewing the scoliotic spine in 3D, the SRS has appointed a 3D Scoliosis Committee to evaluate the impact of 3D analysis of the scoliotic spine and to establish a 3D classification of scoliosis [30] using a database of 600 AIS spine reconstructions and mathematical clustering techniques to determine curve patterns. The final 3D classification will become available in the coming years, and will be applicable to both CT-scan and EOS^®^ 3D reconstructions.

Due to its ability to provide a full-body view, the EOS^®^ system has been used in some studies to assess sagittal malalignment in different pathologies as well as its relationship with the cervical spine and the lower limbs.

#### Cervical spine and head in sagittal balance assessment

EOS^®^ was used by Le Huec et al. to study cervical sagittal balance in a group of 106 asymptomatic individuals [31], where parameters of cervical sagittal balance were defined for the first time by analogy to pelvic and thoracolumbar spinal sagittal balance.

#### Spine, pelvis, and lower limb sagittal balance

With the advent of the EOS^®^ system, it became possible to study the relationships of the femur and the hip joint with the spine through the pelvis. This possibility was stressed by Lazennec et al. [32]. The authors studied different radiological parameters of 46 normal patients (92 hips) in the normal standing position and with one foot on a step (each side alternately) in order to assess the sagittal balance in different postural positions [33]. They concluded that EOS^®^ imaging is a good technique for assessing global spinal sagittal balance and its relationships with the pelvis and the lower limbs. EOS^®^ images also facilitate the screening of patients at risk of posterior impingement in total hip arthroplasty when there is a sagittal malalignment. This area of study is still in its infancy, but its full potential is about to be exploited due to the ability of the EOS^®^ system to provide a global view of the spine and the lower limbs.

**Fig. 4:**
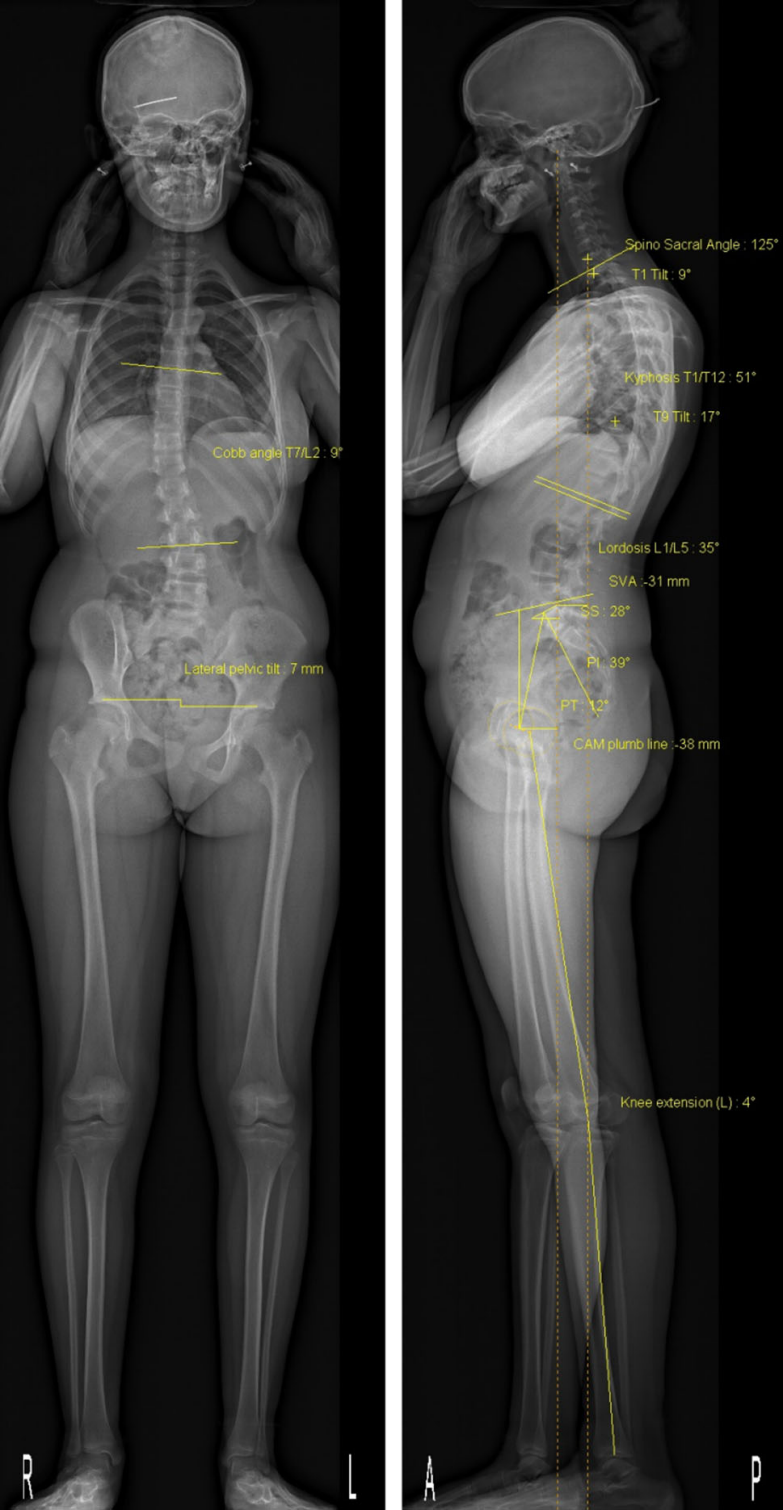
Example of posture analysis in a single setting using the EOS^®^ system

A new 3D Posture software package is provided with the new version of the EOS^®^ software; this software permits the calculation of several parameters related to the posture of the subject in both 2D and 3D. (Fig. 4).

### Degenerative spine

Rillardon et al. compared discography (after the injection of intradiscal contrast solution) by EOS^®^ imaging to MRI for the study of intervertebral disc spaces [34]. The authors showed that 39 % of disc spaces were narrowed, with high correlations between the two imaging modalities for the detection of narrowing (*p* = 0.008) as well as for the determination of the degree of narrowing (*p* = 0.02). The anterior and posterior margins of the intervertebral discs were visible when EOS^®^ was used in 22 and 64 % of cases, respectively, as compared to 84 and 97 % when MRI was used. There were nine cases of disc herniation on MRI, none of which were detected by EOS^®^ discography. EOS^®^ imaging is therefore not a good alternative to MRI for the study of degenerative disc disease.

### Vertebral osteoporosis

EOS^®^ imaging was also tested for the study of vertebral osteoporosis. It was first compared to dual-energy X-ray absorptiometry (DXA) using a Hologic^®^ device for measurement of bone mineral density (BMD) [35]. Both techniques were used to determine BMD on the European Spine Phantom, and the results were compared to the values given by the manufacturer of the phantom. EOS^®^ imaging was more accurate than Hologic^®^ (5.2 % compared to 7.2 %) and had very good reproducibility [35]. In another study, it was used to determine the BMDs of 14 fresh-frozen vertebrae, as well as to provide a 3D reconstruction of these vertebrae [36]. Thus, a subject-specific finite element model (FEM) of each vertebra was created, which led to better prediction of the failure load of these vertebrae.

### Axial spondyloarthritis

EOS^®^ 2D was compared to conventional radiography (CR) during the follow-up of patients with axial spondyloarthritis [37]. It was found to be equivalent to CR when assessing ankylosis on dynamic views, but there was less agreement in the diagnosis of sacroiliitis; interpretation was more difficult with the EOS^®^ system. The authors concluded that EOS^®^ imaging could be used during patient follow-up, but not for the diagnosis of sacroiliitis.

## Lower limb and pelvis

### Accuracy, reproducibility, and reliability of EOS measurements

#### Pelvis

*Validation for pelvis measurements* A cadaveric study compared conventional and EOS^®^ radiographs (both 2D) for pelvis imaging, with different degrees of sagittal tilt and axial rotation (−15° to 15°), of a human cadaveric model [38]. Six different measurements were made on each radiograph, as well as the presence of coxa profunda and crossover sign. Intra- and interobserver reproducibility were high for both techniques (intraclass coefficients of 0.795–1.000). Intra- and interobserver agreement on the presence of coxa profunda and crossover sign was also high. There was a high correlation between the two techniques, but there was a significant difference between the linear measurements (probably because of the magnification effect in conventional radiography). Rousseau et al. evaluated the use of the EOS^®^ 2D/3D imaging system for the assessment of axial rotation of the pelvis [39]. They measured the axial rotation of a dry pelvis by EOS^®^ imaging and compared the result to that given by a laser line reference goniometer, and found EOS^®^ imaging to be reliable (inter- and intraobserver reliability of 0.33° and 0.23°, respectively) and accurate (−0.39°, SD 0.77°). The sterEOS^®^ software allows 3D measurements of the pelvis but does not, as yet, offer a 3D reconstruction. Full 3D reconstruction of the pelvis and the acetabulae is still being developed; it is not available with the commercial software [40, 41].

**Fig. 5:**
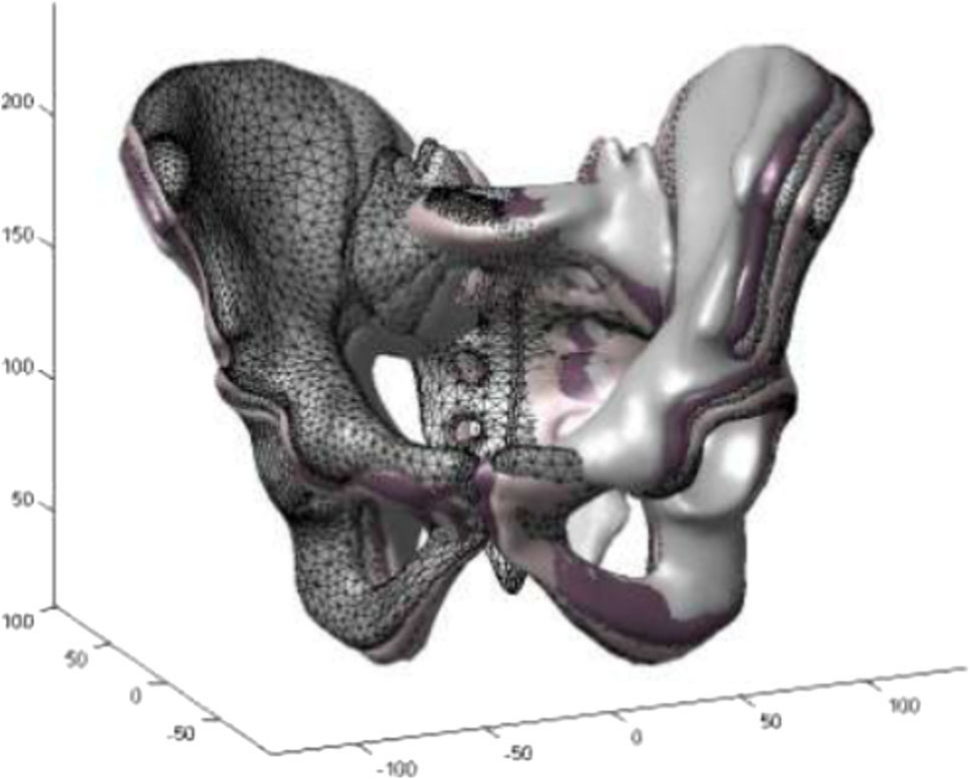
Superposition of 3D reconstructions obtained by the sterEOS^®^ research software for 0°, 5°, 10°, 15°, and 20° of axial rotation for the same pelvis [42]

In a recent study, the effect of patient malpositioning in the EOS booth was studied using six adult pelvises. 3D reconstruction of the pelvis using EOS^®^ was proven to be valid and reliable even with 15º of axial rotation of the patient during X-ray acquisition (Fig. 5) [42].

*Obstetric pelvimetry* Sigmann et al. used EOS^®^ imaging for obstetric pelvimetry, and compared it to CT scan and direct manual measurement on ten cadaveric pelvises [43]. The evaluated parameters were the obstetric conjugate diameter (OCD), the true conjugate diameter (TCD), the median transverse and transverse diameters (MTD and TD), the intertuberous diameter (ITD), the interspinous diameter (ISD), the anteroposterior diameter (APD) of the pelvic outlet, and the Magnin index (OCD + MTD). There was excellent correlation between EOS^®^ imaging and CT scan or manual measurements for all parameters except for the ISD, which was underestimated on EOS^®^ imaging by 4.9 mm.

#### Lower limb

Guenoun et al. evaluated the inter- and intraobserver reliability of EOS^®^ 2D/3D imaging for lower extremity measurements (femur length, tibia length, lower limb length, HKS angle, HKA angle, femoral offset, neck shaft angle, femoral head diameter, femorotibial rotation, tibial torsion, femoral neck length, flexum/recurvatum, femoral anteversion) [44]. The authors compared values obtained by EOS^®^ 3D reconstruction with values obtained by 2D EOS^®^ X-rays. They found that both methods gave high inter- and intraobserver reliability, with slightly better results obtained from 3D reconstruction. However, this technique was not compared to CT scan (the established gold standard for torsion and rotation), and the population consisted of 25 patients admitted for total hip arthroplasty, so it is not representative of the normal population.

In order to evaluate EOS^®^ 3D reconstruction for the assessment of limb length, Escott et al. measured the length of a phantom femur 10 times with CT scanogram (scout view), conventional radiography, and EOS^®^ slow and fast (ultralow dose), and compared these results to the known length of the femur [45]. They found EOS^®^ slow and fast to be more accurate than both CT scanogram and conventional radiography (*p* < 0.0001). EOS^®^ slow and fast both showed excellent measurement reproducibility (ICC > 0.9). The radiation dose was lower with EOS^®^ fast than with the three other modalities.

In a study comparing EOS^®^ imaging with computed tomography (the gold standard) for measuring femoral and tibial rotational alignment [46], 43 lower limbs were retrospectively reviewed in 30 patients who had both EOS^®^ radiographs and CT scans performed as part of the workup of their pathology. Both techniques had excellent interobserver reproducibility. There was no significant difference between measurements by the EOS^®^ system or CT scan (*p* = 0.5 for femoral torsion and *p* = 0.4 for tibial torsion), and the authors concluded that EOS^®^ imaging is a good alternative to CT scan for the evaluation of femoral and tibial torsion.

A similar study was performed on cadaveric femurs in different axial rotations (−10°, −5°, 0°, 5°, and 10°), and the femoral torsion measurements obtained from EOS^®^ 3D reconstruction were compared to those gained from CT-scan 3D reconstruction [47]. The interobserver reproducibility and the intraobserver repeatability were excellent for both CT-scan and EOS^®^ 3D reconstructions (ICC 0.981–0.998), and there was no statistically significant difference between the values yielded by these two techniques and the reference values of femoral torsion.

**Fig. 6:**
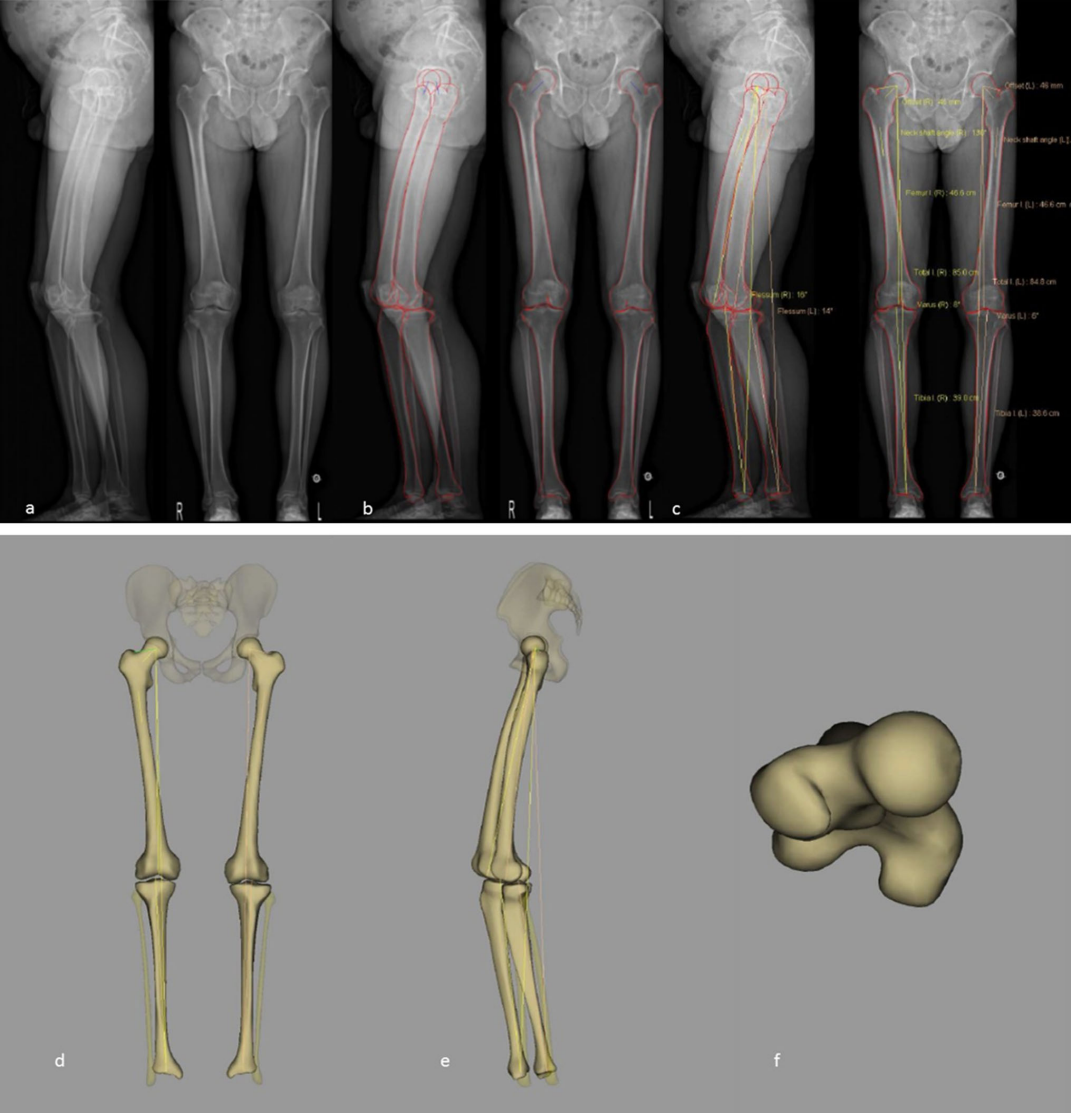
Example of a biplanar radiograph (**a**), with subsequent bony contour determination (**b**), measurements (**c**), and 3D reconstruction (**d–e**)

An example of 3D reconstruction of the lower limbs is shown in Fig. 6.

#### Foot and ankle

Rungprai et al. evaluated the validity and accuracy of radiological parameters for the foot and ankle measured on EOS^®^ 2D radiographs as compared to the corresponding results obtained from conventional radiographs [48]. They measured ten foot and ankle and eight lower-limb radiographic parameters on 65 conventional images, and EOS^®^ 2D was performed in two stances: staggered and nonstaggered feet. Measurements from EOS^®^ 2D in both positions were found to be repeatable (ICC > 0.938), reproducible (ICC > 0.927), and accurate, with no statistically significant difference between EOS^®^ and conventional radiography observed for the ten foot and ankle parameters except in the staggered position, where a difference in limb length measurements for the rear leg was observed (probably due to the magnification effect).

### Pediatric lower limb and pelvis

In order to assess EOS^®^ 3D measurement of the lower limbs in children and adolescents, Gheno et al. compared 3D EOS^®^ and 3D CT-scan reconstructions of eight dried bones in three different axial rotations (−10°, 0°, and +10°) and found no difference between the two techniques in the measurement of femoral length, tibial length, femoral mechanical angle, tibial mechanical angle, frontal knee angulation, lateral knee angulation, and femoral neck–shaft angle [49]. They repeated these measurements using the EOS^®^ system in 27 children and adolescents (age 11–17) and found a difference between EOS^®^ 2D and 3D measurements of tibial length (*p* = 0.003), femoral mechanical angle (*p* < 0.001), and femoral neck–shaft angle (*p* = 0.001) but no difference for any other angle. The interobserver agreement was excellent for femoral length, tibial length, frontal knee angulation and lateral knee angulation, and moderate for femoral mechanical angle, tibial mechanical angle, and femoral neck–shaft angle.

Assi et al. evaluated EOS^®^ 3D reconstruction for five asymptomatic and five cerebral palsy children aged 5–15. They found reconstruction to be feasible even during growth and in the presence of an open physis [50]. The authors also showed excellent reproducibility for all parameters (tibial mechanical angle, femoral tibial rotation, neck–shaft angle, femoral mechanical angle, femoral physiological angle, femoral torsion and tibial torsion, femoral tibial mechanical angle, femoral length, and tibial length).

EOS^®^ imaging was also used by Gaumétou et al. to measure femoral and tibial torsion in 114 healthy volunteers aged 6–30 years [51]. Measurements obtained using the EOS^®^ system showed good reproducibility (ICC 0.82 and 0.84 for tibial torsion and femoral torsion, respectively) and good to excellent repeatability (ICC ranging from 0.79 to 0.96).

### Total hip arthroplasty

#### Acetabular and femoral component positioning and orientation

EOS^®^ was compared to conventional X-rays and CT scan for the study of implant positioning (acetabular and femoral) in total hip arthroplasty (THA), and was deemed either equivalent or superior to previous imaging modalities [52–55]. Furthermore, it allows the study of implant positioning and orientation in multiple stances (sitting, squatting, standing), which is potentially useful when attempting to understand the bad results obtained in some patients who appear to have well-positioned implants on CT scan or X-ray (taken in supine or standing positions) [56, 57].

#### Computer-navigated total hip arthroplasty

**Fig. 7:**
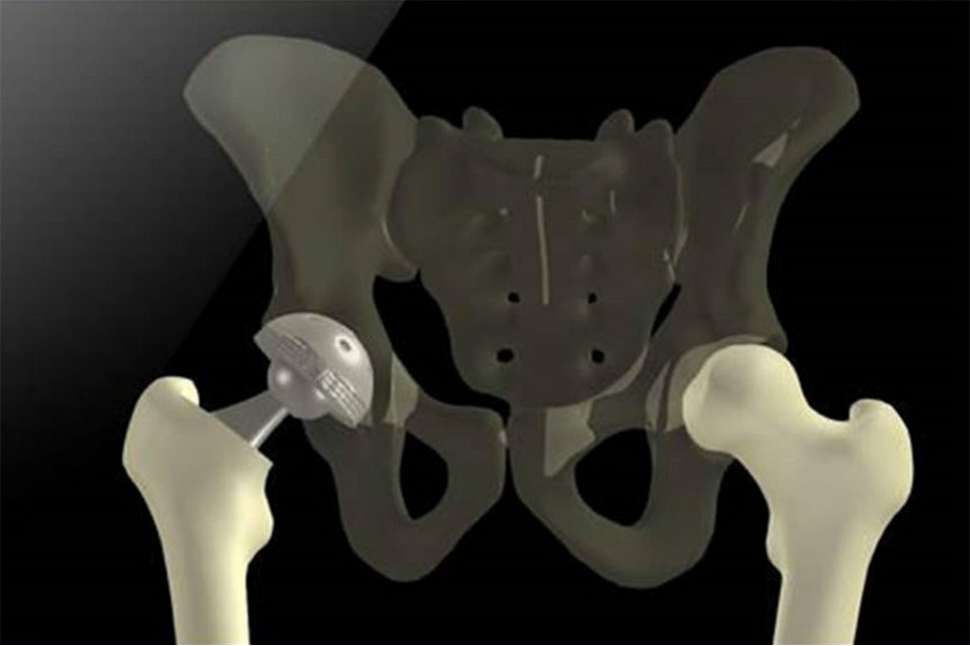
hipEOS^®^ simulation of THA

EOS^®^ imaging also allows computer-navigated THA [58, 59], especially since the implementation of the hipEOS^®^ software, which allows 3D simulation of different sizes and orientations of implants (Fig. 7).

#### Case of patellofemoral pain explained by EOS^®^ imaging

In a case report of patellofemoral pain following total hip replacement, EOS^®^ 2D/3D was found to be superior to conventional radiography and CT scan in demonstrating the abnormal postural adaptation of hip flexion and internal rotation that the patient used to compensate for postoperative limb length discrepancy [60]. It also showed the lateral subluxation of the patella that resulted from the aforementioned postural adaptation and caused his patellofemoral pain. According to the authors, no other technique was able to detect this abnormal posture, and the patient recovered after revision of her total hip replacement and correction of the limb length discrepancy.

### Total knee arthroplasty

The EOS^®^ imaging technique was used in TKA to determine the best landmarks for studying implant positioning [61], and was validated for knee axis measurement after TKA [62].

### EOS^®^ imaging in biomechanical engineering

#### Gait analysis using external markers

Südhoff et al. compared three external attachment systems that are used to minimize soft-tissue artifacts when studying bone motion during gait analysis [63].

#### Joint motion models

Azmy et al. developed a new experimental setup for the cadaveric study of knee joint kinematics that combined EOS^®^ 3D reconstruction with an optoelectronic motion capture system [64]. This experimental setup was reliable for both translation and rotation measurements, and could in the future serve as a basis for knee implant evaluation as well as validation of the finite-element-based models of the patellofemoral joint. Jerbi et al. described a new method based on EOS^®^ imaging for in vivo kinematic studies using an initial 3D reconstruction and subsequent radiographs in other positions, from which they estimated joint motion [65, 66].

#### Determination of the hip joint center using 3D EOS^®^

Pillet et al. assessed the reliability and accuracy of the EOS^®^ system when determining the hip joint center (HJC) with relation to external pelvic markers [67], which is essential for gait analysis. Other reported techniques are either less accurate (3D ultrasound), require more ionizing radiation (roentgen stereophotogrammetric analysis, RSA), or cannot determine HJC in relation to the pelvic coordinate system since the patient is supine (MRI). Sangeux et al. assessed [68] hip joint center localization techniques that are employed to analyze gait in an adult population using EOS^®^ imaging as an image-based reference. The same study was then applied by Assi et al. to children with cerebral palsy and normal controls [69].

#### Body segment parameters

Dumas et al. used EOS^®^ 3D reconstruction to determine body segment parameters (BSPs) of human thigh in eight males and eight females [70]. BSPs are useful for biomechanical analysis of human movement and posture in sports, ergonomics, rehabilitation, and orthopedics. They compared the results of this method to the results of predictive equations determined by previous cadaveric analysis, gamma-ray scan, DEXA scan, and MRI. They found the EOS^®^ technique to be a good alternative to other methods, and it enabled personalized BSP determination in special populations for whom equations are not available, such as hemiplegics and obese persons.

## Limitations

Although EOS^®^ imaging is currently considered by many users and potential users to be the future gold standard of X-ray imaging of the skeleton due to its known advantages (mainly 3D reconstruction with a low radiation dose), it is still lacking in many aspects. Using the software and technology currently available, diagnosis is improved in only a few patients; only a small fraction of those patients will have their treatment altered, and even fewer will have their outcomes altered because of their change in treatment. There are still many uncertainties at each step of the method, from patient positioning to full 3D reconstruction and angular and distance measurements:

- The image may be wavy if the patient is unable to stand or sit steadily during X-ray imaging, which means that the technique has limited applicability to patients with underlying neurologic or neuromuscular disorders.
- The 2D images on X-ray films present less contrast and therefore suffer from decreased brightness when compared to those provided by conventional digital radiography. However, this is drastically improved when the image contrast is modified on a computer screen.
- 3D reconstruction is semi-automatic, which means that the X-ray operator adjusts the shape of the standard bone segment given by the software to make it patient specific. This may increase the risk of error.
- Available software packages do not allow 3D reconstructions for children below the age of 5–6 years, because they were originally conceived for adult bones.
- 3D reconstruction of the patella is still impossible, and so is 3D reconstruction of the rib cage and congenital anomalies of the spine; a dedicated software package is still under patent.
- 3D angular measurement of severe deformities of the limbs is impossible due to the use of a statistical model based on “normal” bones. Any 3D reconstruction of a severe deformity could lead to biased measurements.
- 3D reconstruction involves just the outer bone surface (“envelop”); the inner structure or architecture of the bone is not considered because the reconstruction is based on only two radiographies, unlike the CT scan, where many acquisitions are performed to generate an axial view. Therefore, EOS is comparable to CT scan only for 3D reconstruction of the bone envelop, but it has the advantage of providing much less radiation to the patient than CT does.

## Cost effectiveness and impact

The basic cost of the EOS^®^ machine with its corresponding software for acquisition, 2D processing, and 3D reconstruction is around 500,000 euros. The implementation of such an imaging system requires clear-cut advantages compared to other imaging modalities, not only from the physician’s point of view. Thus, Dietrich et al. compared the radiation dose, examination time, patient comfort, and the financial break-even point of EOS^®^ 2D imaging and standard radiography [71]. They found EOS^®^ 2D to have a lower radiation dose than standard radiography (158.4 ± 103.8 vs 392.2 ± 231.7 cGy cm^2^) and a shorter mean examination time (248 vs 449 s). On the other hand, digital radiography had higher patient comfort regarding noise and a lower break-even point (2602 radiographs/year, compared to 4077 radiographs/year). Given that radiation exposure reduction was the only proven health benefit derived from the use of EOS^®^ imaging [72], Faria et al. did a cost-effectiveness analysis quantifying the health benefits from reduced radiation exposure [73], following a study for the Health Technology Assessment (HTA) program of the British NHIR [72]. They created a model that estimated the loss of quality-adjusted life years (QALY) due to cancer that was attributable to a lifetime’s worth of radiation exposure resulting from the diagnosis and long-term monitoring of the major indications of both standard X-ray and EOS^®^ imaging. The authors concluded that EOS^®^ imaging was not cost-effective because the loss of QALY attributable to cancer secondary to radiation exposure from standard X-ray imaging is already small. For EOS^®^ imaging to be considered cost-effective, it must either provide a throughput of a minimum of 15,100/year (60 patients/day for 251 working days), or prove itself superior to other imaging modalities in terms of patient-oriented clinical outcomes, i.e., it should lead to better outcomes, not just better image quality.

## Conclusion

EOS^®^ imaging is certainly a very useful tool for research and is gradually replacing conventional digital radiography in clinical settings due to the low radiographic exposure associated with this technique and the proven benefits of using it for diagnosis and during treatment follow-up. Many studies using EOS^®^ imaging, sometimes in association with other imaging modalities or gait analysis, and with very optimistic aims (better diagnosis, treatment planning, and outcome), are currently being undertaken or are still being designed by research teams. Numerous improvements to this technique are still on the way, due to the work of its founders and research teams worldwide, and we have no doubt that EOS^®^ imaging will rapidly increase in popularity during the coming years in both the research and clinical settings, and the number of publications dealing with EOS^®^ imaging will dramatically increase in years to come.
